# Functionalized Activated Carbon Derived from Palm Kernel Shells for the Treatment of Simulated Heavy Metal-Contaminated Water

**DOI:** 10.3390/nano11113133

**Published:** 2021-11-20

**Authors:** Rabia Baby, Mohd Zobir Hussein, Zulkarnain Zainal, Abdul Halim Abdullah

**Affiliations:** 1 Material Synthesis and Characterization Laboratory, Institute of Advanced Technology, Universiti Putra Malaysia, Serdang 43400, Selangor, Malaysia; rabia.Shaikh@iba-suk.edu.pk (R.B.); zulkar@upm.edu.my (Z.Z.); halim@upm.edu.my (A.H.A.); 2 Department of Education, Sukkur IBA University, Sukkur, Sindh 65200, Pakistan

**Keywords:** heavy metals, activated carbon, metal adsorption, water contamination, and water purification

## Abstract

Heavy metal contamination in water poses a great risk to human health as well as to the lives of other creatures. Activated carbon is a useful material to be applied for the treatment of heavy metal-contaminated water. In this study, functionalized activated carbon (FAC) was produced by the induction of nitro groups onto activated carbon using nitric acid. The resulting material was characterized in detail using the XRD, Raman, BET, FTIR, and FESEM techniques. The FAC was used for the treatment of heavy metal-contaminated water using different adsorption parameters, i.e., solution pH, contact time, adsorbent dosage and heavy metal ion concentrations, and these parameters were systematically optimized. It was found that FAC requires 90 min for the maximum adsorption of the heavy metal ions; Cr^6+^, Pb^2+^, Zn^2+^ and Cd^2+^. The kinetic study revealed that the metal ion adsorption follows the pseudo-second-order. The Freundlich and Langmuir isotherms were applied to determine the best fitting adsorption isotherm models. The adsorption capacities were also determined for each metal ion.

## 1. Introduction

The industrialization has made life easier; unfortunately, at the same time, it has contributed significantly to environmental pollution, i.e., water pollution, soil pollution and air pollution [1,2,3]. Because of increased industrialization, clean and pure drinking water resources are becoming scarcer day by day. There are many sources of water contaminants; bacteria, viruses, organic compounds, organic dyes, and heavy metal ions such as cadmium, chromium, zinc, arsenic and mercury.

Heavy metal contaminants are non-biodegradable, causing immense risk to humans and other living creatures. These heavy metals accumulate in the body and harm health, such as through kidney damage, cancer, hepatitis, anemia, miscarriages, nephritic syndrome and encephalopathy, etc. [4,5,6]. There are various sources of heavy metals. Lead (Pb^2+^) enters into the environment from metal mining industries, lead-acid batteries, glass, paper, and polishing industries. Cadmium (Cd^2+^) enters the water from electroplating, batteries and photovoltaic cells, fabric factories and metal [7,8,9,10]. Chromium (Cr^6+^) causes liver damage, stomach distress and nephritis, and is also responsible for nasal mucous ulcers [11,12]. Zinc (Zn^2+^) enters the water from mining activities and industrial waste. Zinc (Zn^2+^) causes several adverse health effects, e.g., gastrointestinal problems and cardiovascular problems, and can cause DNA damage, resulting in carcinogenic effects and neurotoxicity [13].

Heavy metal ions can be separated from water in several ways: the ion exchange method, precipitation, coagulation, the biosorption method, and the adsorption method [5,14,15,16,17]. Adsorption is a process in which the chemicals are accumulated on a solid (adsorbate) surface, and the reverse of this process, where the adsorbed chemicals are detached from the adsorbate, is known as desorption. The removal of heavy metals from water by the adsorption process offers many advantages over other methods, e.g., it is an economical process, it is user-friendly, it has high efficiency, it regenerates the adsorbate, and the adsorbate can easily be regenerated and reused [1,2,8,10,18].

The process of adsorption is affected by various parameters; the dosage of the adsorbate, the solution pH, the concentration of the metal ions and the contact time, etc. [4,5,18]. The increase in the dosage amount of the adsorbate rapidly increases the removal efficiency due to the more significant number of sites available for the attachment of the adsorbent [19]. The pH of the solution has a more significant impact on the adsorption, and the pH is optimized for the maximum adsorption of different adsorbents. Adsorption has many applications, e.g., the treatment of wastewater treatment, gas/air purifier masks, the purification of oil/petroleum products, the separation of gases, the purification of sugar, nitrate adsorption, the softening of water and decolorization, etc. [20].

The activated carbon structure comprises three parts: microcrystalline graphite, amorphous carbon and reticular carbon of a single plane. The activated carbon structure is mainly composed of the microcrystalline graphite structure. However, its structure is different from graphite. The microcrystalline structure of activated carbon possesses interlayer spacing of 0.34–0.35 nm. It cannot be transformed into graphite even if it is heated higher than 2000 °C; that is why it is known as non-graphite microcrystal [21,22]. This microcrystalline structure imparts the porous structure to the activated carbon. The pore size of activated carbon can be divided into three categories based on its diameter; micropores (width < 2 nm), mesopores (width 2–50 nm) and macropores (width > 50 nm) [23,24]. The adsorption efficiency of the activated carbon depends on its physical structure, pore size and chemical structure, i.e., the presence of different functional groups incorporated during the treatment of the parent material before the activation in the furnace. The most common activators used are acidic, alkaline and neutral, imparting different functional groups onto the surface of the adsorbents [25,26]. Activated carbon can be synthesized from various materials containing carbon; agricultural wastes, apricot stones, sewage sludge, waste of the newspapers, and different parts of the plants [27].

Malaysia, Indonesia, Nigeria, African countries, Cameron and China produce millions of tons of oil palm agricultural waste. Malaysia is one of the top oil palm producers, with about 4.5 million hectares [4,5]. Palm kernel shell (PKS) is a waste of the oil palm upstream industry, is produced in massive amounts, and is disposed of by burning, resulting in environmental pollution and the greenhouse effect. Researchers are trying to utilize the invaluable waste material products and save environmental pollution [4,5,6,27]. In this study, functionalized activated carbon (FAC) was prepared using PKS as a carbon source. Then, the activated carbon obtained was functionalized by the oxidation with nitric acid followed by washing to remove the excess acid. Furthermore, the resulting FAC was used for the treatment of heavy metal-contaminated water. The prepared FAC was found to be very effective in the purification of heavy metal-contaminated water.

## 2. Materials and Methods

The chemicals used were palm kernel shell (PKS), deionized water with a resistivity of 18 MΩ, phosphoric acid (H_3_PO_4_), stock solutions of Cr^6+^, Cd^2+^, Zn^2+^ and Pb^2+^, and nitric acid (HNO_3_) purchased from Sigma Aldrich (St. Louis, MO, USA).

### 2.1. Instrumentation

The instruments used for the characterization of the samples in this work were an FTIR spectrophotometer (Perkin-Elmer 100 series, Waltham, MA, USA), an XRD (Shimadzu, Kyoto Japan), a Raman spectrometer (WiTec, Ulm, Germany), a field emission scanning electron microscope (FESEM) NOVA NANOSEM 230 model, Denton, TX, USA), an inductively coupled plasma-Optical Emission Spectrometer (ICP-OES), Optima 2100 DV Perkin Elmer and a BET surface area analyzer (Micromeritics Model: Tristar II Plus) Norcross, GA 30093-USA.

### 2.2. Pretreatment, Activation and Preparation of the Activated Carbon

The PKS samples were collected from a local Palm Oil Mill in Dengkil, Selangor. The PKS sample was cleaned with deionized water, followed by drying at 60 °C in an oven. After the drying, the PKS sample was ground to a powder in an automatic blender and subjected to activation by the treatment with phosphoric acid according to the previously reported method [28]. In brief, for the optimization of the activation, about 20 g of the PKS sample was treated with various concentrations of phosphoric acid: 0%, 5%, 10%, 20%, 30% and 40% (*v*/*v*) with constant agitation at 120 rpm for one day. The next day, the sample was filtered and dried in an oven at 60 °C. After that, the sample was subjected to a tubular furnace to prepare the activated carbon under a constant nitrogen gas flow at 150 cm^3^/minute. The activated carbon preparation parameters, temperature and holding time were then optimized. The sample treated with 20% phosphoric acid was found to have the highest surface area [28].

### 2.3. Optimization of the Temperature, Holding Time and Functionalization

The temperature for the synthesis of the activated carbon was optimized by putting 5 g of the 20% phosphoric acid-treated PKS sample in a furnace under different temperatures; 500, 600, 700, 800 and 900 °C, with a holding time of 2 h and a heating rate of 10 °C/minutes under an inert environment of an N_2_ (g) flow. The holding time was optimized by varying the carbonization time; 1, 2, 3, 4 and 5 h. These conditions were applied to all of the phosphoric acid-treated PKS samples, as reported previously [27,28]. The following are the optimized conditions applied to synthesize the activated carbon; phosphoric acid, 20%; temperature, 500 °C; and a 2 h holding time [29,30]. The activated carbon prepared with the optimized conditions was found to have the highest surface area and was further functionalized with nitric acid (HNO_3_). The functionalization was optimized by treating the sample with different concentrations of nitric acid; 10, 15, 20 and 25%.

Surface Area Analysis: In the BET analysis, N_2_ gas was absorbed on the sample surface, and where the pressure was applied, and the volume of the absorbed N_2_ gas was measured. The FAC samples were degassed at 290 °C under vacuum for 9 h before the adsorption–desorption measurements were carried out, and we performed the experimental adsorption–desorption measurements. Then, we applied mathematical models for the surface area and porosity analyses [28].

### 2.4. Batch Experiment Set-Up

#### 2.4.1. pH Optimization

The pH for the adsorption studies was optimized by varying the pH of the solution; 2, 3, 4, 5 and 6. Three other parameters were kept constant; a 100 mL solution of each of the metal ions with a concentration of 40 mg/L, an adsorbent dosage of 0.2 g and a contact time of 2 h.

#### 2.4.2. Adsorption Dosage Optimization

The adsorbent dosages were varied; 0.1 g, 0.15 g, 0.2 g and 0.25 g, and all of the other parameters were kept constant; a 100 mL solution of each of the metal ions with a concentration of 40 mg/L, a contact time of 2 h and a pH of 6.

#### 2.4.3. Optimization of the Metal Ion Concentrations

The metal ion concentrations were optimized by varying them; 10, 20, 30, 40 and 50 mg/L in a 100 mL solution and the other three parameters were kept constant; pH 6, a contact time of 2 h and adsorbent dosage of 0.25 g.

#### 2.4.4. Optimization of the Contact Time

The contact time was optimized by varying it; 15, 30, 60, 90 and 120 min. The other three parameters were set; a pH of 6, a concentration of 40 mg/L in a 100 mL solution and an adsorbent dosage of 0.25 g.

## 3. Results and Discussion

### 3.1. BET Surface Area Analysis

**Figure 1:**
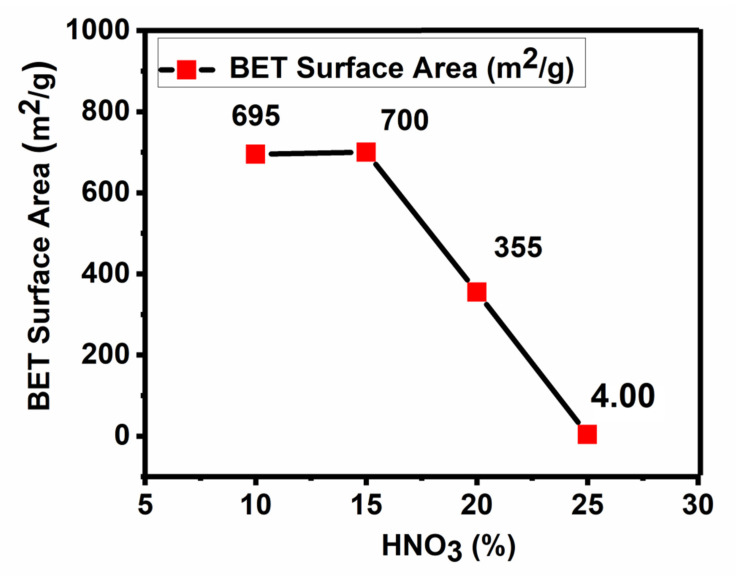
BET Surface area of the FAC prepared at different percentages of nitric acid.

The activated carbon with a surface area of 1099 m^2^/g was selected for the functionalization with percentage concentrations of nitric acid; 10, 15, 20 and 25%. The oxidation with nitric acid resulted in the functionalization of the activated carbon. These FAC samples were subjected to BET analysis to determine their surface area, given in Figure 1. As shown in the figure, the FAC prepared at 15% nitric acid was found to have a maximum surface area of 700 m^2^/g, and this sample was then selected for further parametric optimization.

### 3.2. Adsorption–Desorption Isotherms

**Figure 2:**
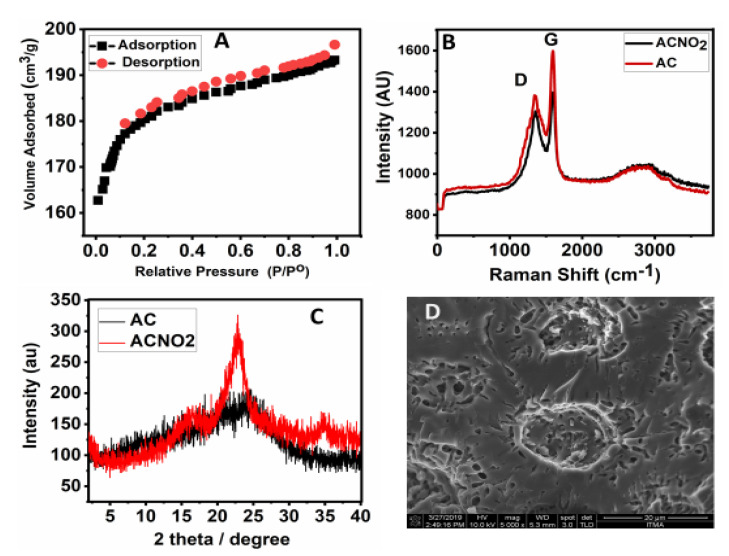
Adsorption—desorption isotherm of FAC (**A**), Raman spectroscopy of AC and FAC (**B**), XRD diffraction patterns of AC and FAC (**C**) and a FESEM micrograph of FAC (**D**).

The isotherms for the adsorption and desorption for the FAC are shown in Figure 2A, which follows Type I by the IUPAC classification. The Type 1 isotherm of the adsorption and desorption suggests the nanoporous nature of a surface area of 700 m^2^/g for the activated carbon treated with 15% HNO_3_. That sample is termed ‘functionalized activated carbon’ (FAC). The isotherm is similar to the other activated carbons reported in the literature [27,28].

### 3.3. Raman Spectroscopic Analysis

Raman spectroscopy is a widely used technique for analyzing different types of carbon-based materials; amorphous carbon, activated carbon, graphite, graphene, graphene oxide and diamond, etc. [29,30]. The Raman bands are associated explicitly with the internal structure; the G band is graphitic sp^2^ hybridized carbon, and the D band is related to the disorders/defects in the graphitic structure [28,31,32]. Figure 2B shows the Raman spectra of AC and FAC, where the D and G bands in AC have higher intensity but are relatively lower in FAC. The I_D_/I_G_ ratio for AC was found to be 0.86 compared to 0.93 in FAC. The data obtained strongly suggest the successful functionalization of AC with the nitrate group [31,32].

### 3.4. X-ray Diffraction and Surface Morphology

The XRD patterns of activated carbon (Figure 2C) show a hump at 2ϴ = 5–35, similar to the one reported in the literature [27,28]. The functionalization of activated carbon with the nitrate group resulted in XRD patterns with relatively higher intensity, possibly due to the nitro group bonded on the AC surface, as shown in Figure 2C. It also suggests that the higher crystallinity of FAC compared to AC. Figure 2D shows the surface morphology obtained by the FESEM technique, showing the porous structure of FAC and a similar structure for activated carbon [4,27,28].

### 3.5. Infrared Spectroscopy

**Figure 3:**
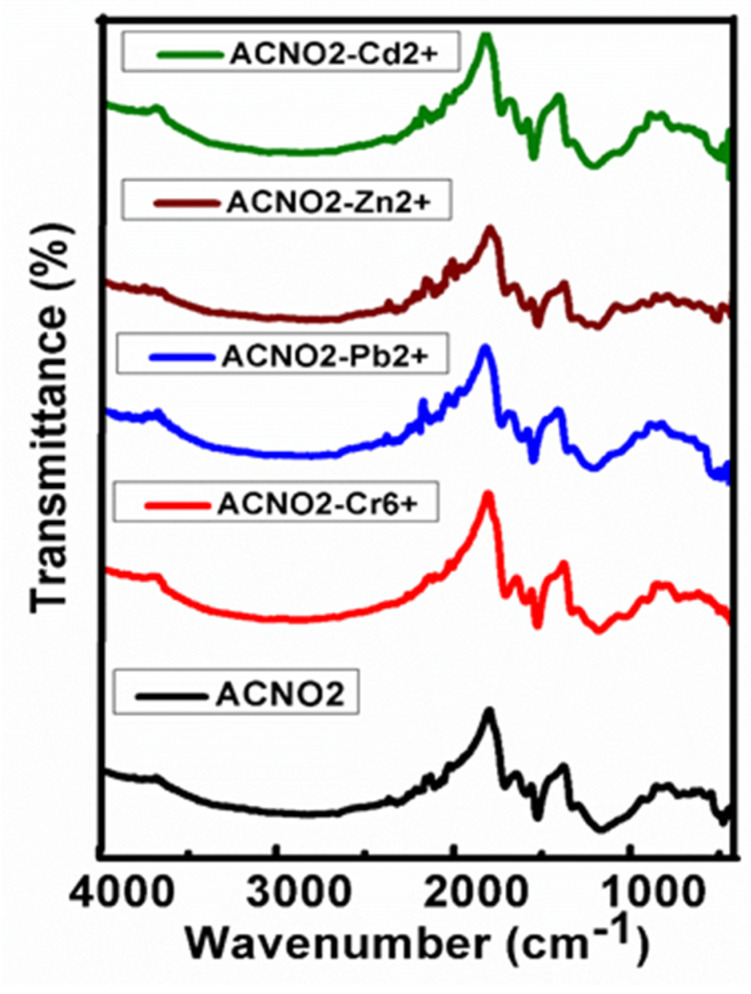
FTIR spectra of FAC before and after the adsorption of the metal ions.

Infrared spectroscopy is a valuable technique for the functional group analysis of the samples of interest. Figure 3 shows the FTIR spectra of the FAC before and after the adsorption of the metal ions; Cr^6+^, Pb^2+^, Cd^2+^ and Zn^2+^. The FAC sample shows the characteristic functional group bands of C-H., CH_2_, C-N, nitro groups and a C-C bond, etc. [33,34]. These infrared bands of the functional group have been shifted, especially for C=O, N=O and N-O bands, as these functional groups provide the active sites for the adsorption of metal ions [4,6,27,28]. The slight shifts in the functional groups’ bands (Table 1) suggest the successful adsorption of the metal ions on the active sites of the FAC. The metal quantification results from the inductively coupled plasma (ICP) analysis also complement the FTIR results for the adsorption of the metal ions.

### 3.6. Batch Studies

#### 3.6.1. Effect of pH

**Figure 4:**
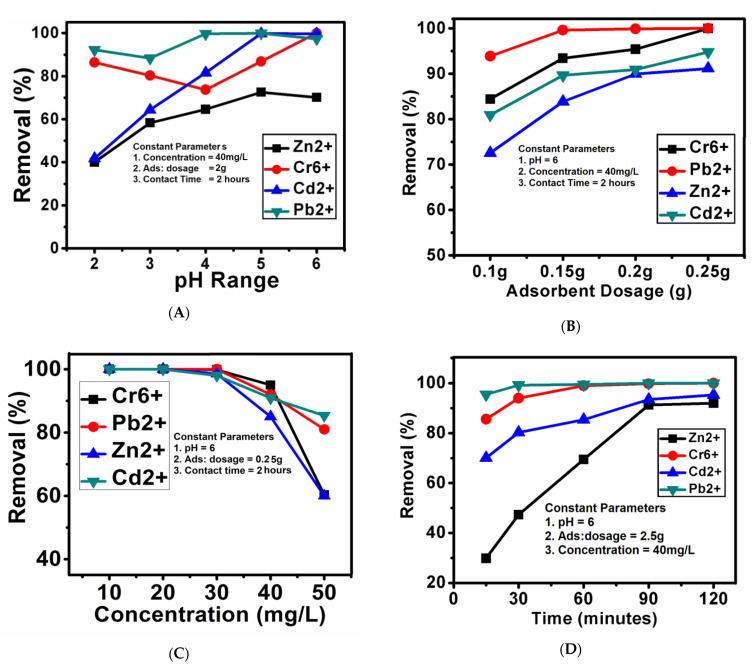
(**A**–**D**) The effects of different parameters; pH, adsorbent dosage, metal ion concentration and contact time on the percentage removal of the metal ions.

The pH is the critical parameter in adsorption studies as it has an immense effect on the interaction of metal ions with adsorbents [35]. In this study, the pH was varied up to 6 because, at a higher pH, metal ions may produce precipitation and adsorption results that are not reliable [35]. The overall adsorption of the heavy metal ions Cr^6+^, Cd^2+^ and Pb^2+^ was found to be increased with the increase in pH, with the maximum adsorption of almost 100% at pH 6, except for Zn^2+^, which showed the highest adsorption of 70% at pH 5, as shown in Figure 4. This optimized pH was then selected for the remaining batch studies; dosage, concentration and contact time.

#### 3.6.2. Effect of the Adsorbent Dosage

Under the optimized pH condition, the adsorbent dosage was studied by varying the amount of the adsorbent dosage (FAC); 0.1, 0.15 g, 0.2 and 0.25 g, in 100 mL of 40 mg/L heavy metal ion solutions. The adsorption of the heavy metal ions was found to be more than 70% for metal ions, which was increased with the increase in the adsorbent dosage. The metal ions Cr^6+^ and Pb^2+^ were 100% removed at the adsorbent dosage of 0.25 g, while the metal ions Cd^2+^ and Pb^2+^ were removed by more than 90%, as shown in Figure 4.

#### 3.6.3. Effect of the Metal Ion Concentration

The effect of heavy metal ion concentration was determined by varying their concentrations. Different concentrations of heavy metal ions; 10, 20, 30, 40 and 50 mg/L were used. All four metal ions were found to be 100% removed even up to 30 mg/L, and the adsorption at 40 mg/L was decreased to 95%, 92%, 90% and 85% for Cr^6+^, Pb^2+^, Cd^2+^ and Zn^2+^, respectively. At the maximum concentration of 50 mg/L, the adsorption was about 85% for three metal ions; Cr^6+^, Pb^2+^ and Cd^2+^. However, the Zn^2+^ metal ion adsorption was found to be around 60%. Figure 4 shows the effect of concentration on the adsorption process. Similar adsorption trends for these metal ions have also been reported previously [4,27].

#### 3.6.4. Effect of Contact Time

The effect of contact time on the adsorption process was determined by varying the contact times; 15, 30, 60, 90 and 120 min under the previously optimized parameters. The Pb^2+^ and Cr^6+^ ions showed quick adsorption as more than 80% adsorption took place in the first 15 min and raised to 100% within 60 min. Cd^2+^ ions showed better adsorption starting with 70% at 15 min and gradually increasing to above 90% within 120 min. However, Zn^2+^ ions took more time for adsorption compared to all of the other three metal ions. They started from 30% initially at 15 min and gradually reached 90% within 120 min. The overall adsorption increased with the increase in contact time between the metal ion solution and the adsorbent.

Previously work on the preparation of activated carbon using PKS materials (non-functionalized activated carbon) as the precursor was reported by Nicholas et al. (2018). The non-functionalized activated carbon was able to remove heavy metal ions; Cr^6+^, Pb^2+^, Cd^2+^ and Zn^2+^ from the simulated aqueous solution up to concentrations of 15 mg/L [27]. In another study, Mohibullah et al. (2020) synthesized activated carbon from Albizia lebbeck and Melia azedarach trees and applied them to remove Pb^2+^ and Cd^2+^ metal ions from an aqueous solution [36]. They found that the activated carbon of Albizia lebbeck trees removed 75% of the Pb^2+^ and 77% of the Cd^2+^ from a 100 mL solution with a concentration of 40 mg/L. They also applied the activated carbon of the Melia azedarach tree to remove these two metal ions under the above identical conditions. They found that the activated carbon removed 62% and 66% of the Pb^2+^ and Cd^2+,^ respectively [36].

Shahrokhi et al. (2021) synthesized activated carbon from pulverized tire waste and used it to remove Pb^2+,^ Cu^2+^ and Zn^2+^ metal ions. The adsorption capacities were found to be 322.5, 185.2 and 71.9 mg.g^−1^ for Pb^2+^, Cu^2+^ and Zn^2+^, respectively. Furthermore, the adsorption was fitted well with the pseudo-second-order kinetics [37]. Zaini et al. (2021) activated the pore texture of fiber-based activated carbon and applied it to remove Cu^2+^ and Pb^2+^. The material was able to remove 50% of the Cu^2+^ and 75% of the Pb^2+^ from a 50mL aqueous solution with a concentration of 20 mg/L Cu^2+^ and 40 mg/L Pb^2+^ [38]. Vishnu et al. (2020) impregnated activated carbon with biosynthesized melanin and applied it to remove heavy metals; Hg^2+^, Cr^6+^, Pb^2+^ and Cu^2+^ from an aqueous solution [39]. The melanin-impregnated activated carbon was found to removed 84.59% Hg^2+^, 86.6% Cr^6+^, 91.1% Pb^2+^ and 93.8% Cu^2+^, bypassing the 5 mg/L heavy metal solution from the column packed with melanin-impregnated activated carbon [39]. Li et al. (2018) p thiol-functionalized activated carbon from sewage sludge and used it for heavy metal removal from aqueous solutions. The synthesized thiol-functionalized activated carbon showed adsorption capacities for Cu^2+^ Pb^2+^, Cd^2+^ and Ni^2+^ of 238.1, 96.2, 87.7 and 52.4 mg/g, respectively. Furthermore, the Langmuir model was fitted well with the adsorption of these metal ions [40]. The functionalized activated carbon designed in this study showed a better removal percentage for Cr^6+^, Pb^2+^, Cd^2+^ and Zn^2+^. Moreover, the kinetics and isotherm models are described in detail in this study.

### 3.7. Kinetics of the Metal Ion Adsorption

The kinetic studies of the heavy metal ion adsorption on the functionalized activated carbon were determined by applying different kinetics models, namely the pseudo-first-order, pseudo-second-order and parabolic diffusion.

The pseudo-first-order equation, in its linear form, is written as follows:

ln (q_e_ − q_t_) = lnq_e_ − k_1_t (1)

where q_e_ represents the adsorption equilibrium, q_t_ is adsorption at any time t, and k is the rate constant that can be determined by obtaining the slope by plotting ln (q_e_ − q_t_) versus t.

The linear equation for the pseudo-second-order can be written as follows:

t/qt = 1/k_2_q_e_^2^ + t/q_e_ (2)

The equation for parabolic diffusion is written as follows:

1 − (M_t_/M_o_)/t = Kt^−0.5^ + b (3)

where the adsorption at time 0 and at time t is represented by M_o_ and M_t_, respectively, in the above equations [4,41,42]. Table 2 contains the detailed parameters of all three kinetic adsorption models.

**Figure 5:**
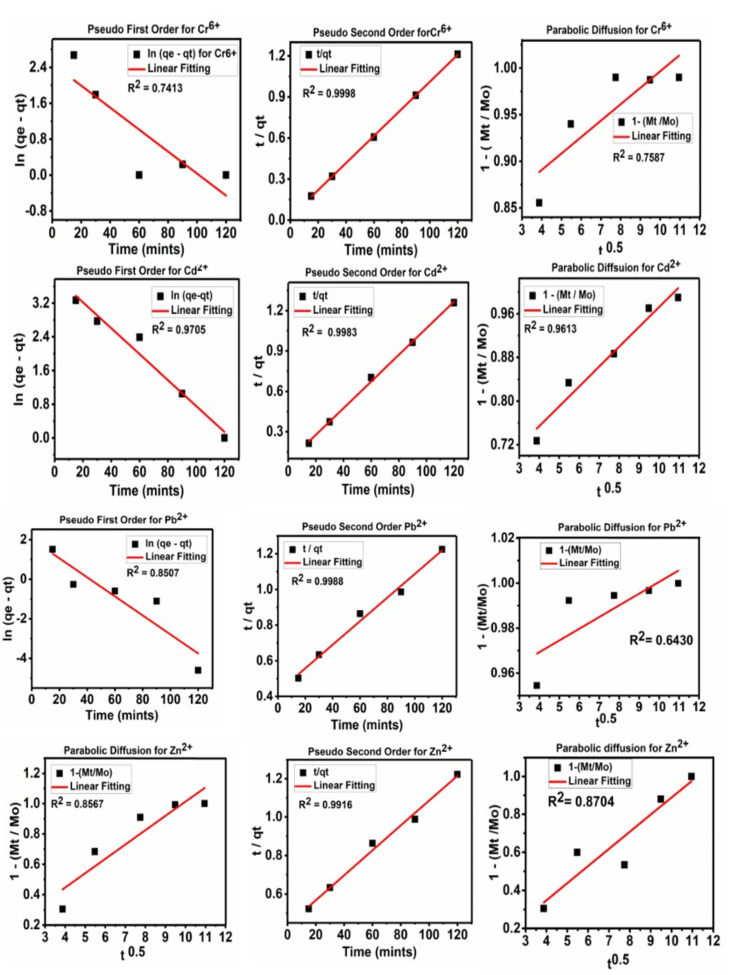
The kinetic fitting for the pseudo-first-order, pseudo-second-order and parabolic-diffusion models for the adsorption of the heavy metal ions; Cr^6+^, Cd^2+^, Pb^2+^ and Zn^2+^ on FAC.

Figure 5 shows the kinetic fitting for the adsorption of heavy metal ions on FAC using the pseudo-first-order, pseudo-second-order and parabolic diffusion models. The adsorption of the heavy metal ions; Cr^6+^, Cd^2+^, Pb^2+^ and Zn^2+^ on FAC were found to follow the pseudo-second-order model. The correlation coefficient, R^2^ was found to be 0.9999 for the pseudo-second-order fitting for the adsorption of heavy metal ions on FAC. Similar kinetics fittings have been reported in the literature [4,27]. The adsorption process that follows the pseudo-second-order kinetics is said to involve chemisorption interactions [4,30,43,44].

## 4. Adsorption Isotherms

The Langmuir and Freundlich isotherms were applied to determine the interaction between the metal ions; Cr^6+^, Pb ^2+^, Cd^2+^ and Zn^2+^ and the adsorbent, functionalized activated carbon (FAC). Equation (4) represents the Langmuir isotherm followed by Equation (5) for its straight line. Equation (6) represents the Freundlich isotherm, followed by its straight-line Equation (7).

Q_e_ = (b Q_m_C_e_)/(1 + bC_e_) (4)

Linear form

C_e_/q_e_ = (C_e_/q_m_) + 1/bq_m_) (5)

Q_eq_ = k_f_ × C_eq_ × 1/n (6)

Linear form

Log q_e_ = logK_f_ + 1/n × log C_e_ (7)

In the above equations, C_e_ and Q_e_ represent the metal ion concentration (mg/L) at equilibrium and the quantity of the metal ions adsorbed (mg/g) on the adsorbent, FAC. Q_m_ represents the metal ions adsorbed (mg/g) on the adsorbent, FAC, and b is a constant. The terms K_f_ and 1/n are Freundlich coefficients.

**Figure 6:**
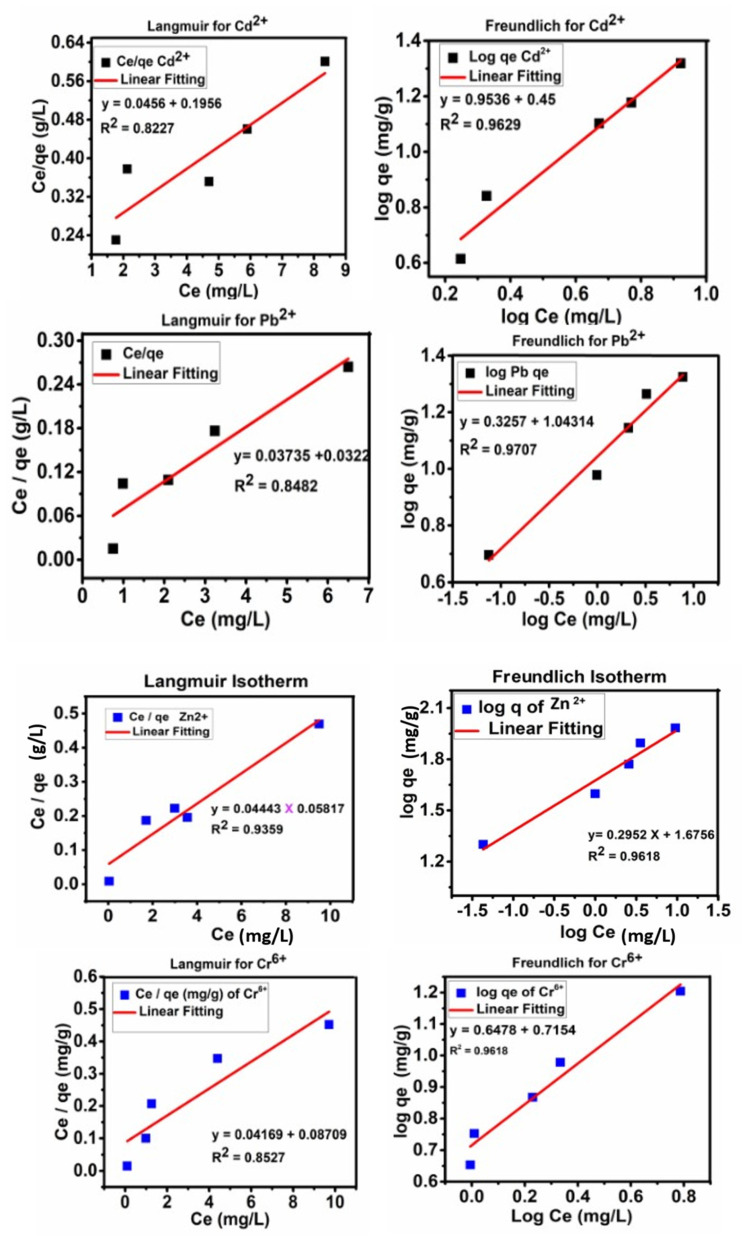
The Langmuir and Freundlich isotherms for the adsorption of Cr^6+^, Cd^2+^, Pb^2+^ and Zn^2+^ on FAC.

Figure 6 shows the fitting of the adsorption for the Freundlich and Langmuir isotherm models. Table 3 shows the correlation coefficients (R^2^) and the values of the constants of the Langmuir and Freundlich isotherms for the Cr^6+^, Cd^2+^, Zn^2+^and Pb^2+^ metal ion adsorption of FAC. The maximum adsorption for the metal ions Cr^6+^ and Pb^2+^ was found to be 40 mg/g and 38 mg/g, and 37 mg/g for both Cd^2+^and Zn^2+^, respectively. The isotherm analysis revealed that the adsorption of Cr^6+^, Cd^2+^, Zn^2+^and Pb^2+^ follow the Freundlich isotherm model, as their correlation coefficient is higher than that of the Langmuir shown in Table 3. The adsorption that follows the Freundlich models suggests that the active adsorbent sites of FAC are uniformly distributed over the surface and that the metal ions are adsorbed onto the surface [4,35]. The Freundlich n_f_ constant values for the metal ion adsorption were found to be between 0.1 and 1, which suggests the favorable adsorption of metal ions on FAC.

## 5. Adsorption Capacity

**Figure 7:**
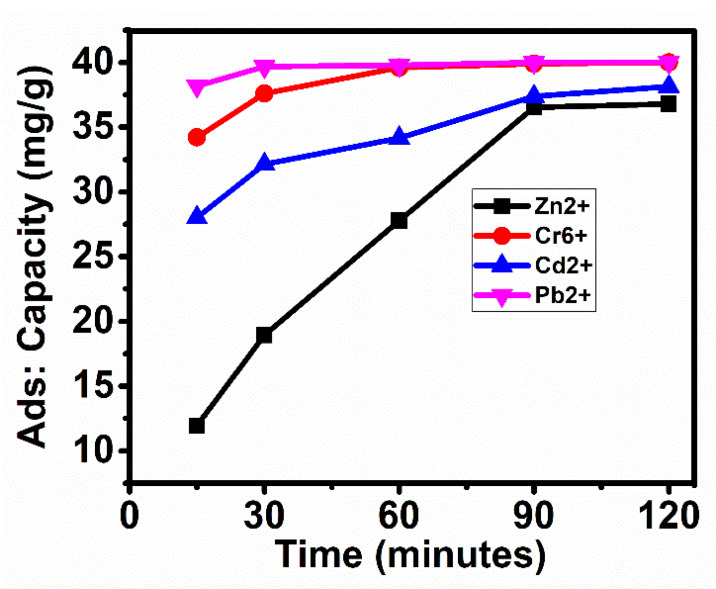
Adsorption capacities (q_e_) of FAC at different time intervals for the adsorption of metal ions; Zn^2+^, Cr^6+^, Pb^2+^ and Cd^2+^.

The adsorption capacity is the amount of metal ions (mg) adsorbed on the adsorbent, FAC, in grams over different time intervals. The adsorption capacity was determined by keeping constant the metal ion concentration at 100 mL of 40 mg/L solutions and 0.25 g of the adsorbent, FAC. Figure 7 shows the adsorption capacity of FAC for different metal ions concerning the contact time. It can be seen that Pb^2+^ ions showed faster adsorption, taking only 30 min for the maximum adsorption of 40 mg/g, followed by Cr^6+^, which took 60 min for the maximum adsorption of 40 mg/g. Cd^2+^ ions showed an adsorption capacity starting from 27 mg/g at 15 min and reaching saturation at 38 mg/g in 120 min. The metal ion, Zn^2+^, started from the lowest adsorption of 12 mg/g and gradually reached the maximum adsorption (saturation) at 37 mg/g in 120 min. The maximum adsorption (q_e_) for each metal ion is given in Table 3.

## 6. Conclusions

In this study, functionalized activated carbon was prepared using phosphoric acid as a chemical activator, and nitric acid was used to functionalize the activated carbon. The resulting FAC was found to contain a high surface area of 700 m^2^/g. The BET adsorption-desorption isotherms suggested the porous nature of the AC. The FTIR results suggested the successful functionalization of the activated carbon. The FAC took 90 min for the maximum adsorption of about 100% for Cr^6+^ and Pb^2+^, and about 90% adsorption for the Cd^2+^ and Zn^2+^ with the original concentration of 40 mg/L. The kinetic studies suggested that the adsorption follows the pseudo-second-order model, and the adsorption isotherms revealed that the adsorption follows the Freundlich model. This study is a greener approach to the preparation of a useful functionalized activated carbon (FAC) from agricultural waste, i.e., palm kernel shells, and the resulting FAC can be applied for environmental remediation, i.e., the treatment of heavy metal-contaminated water. Apart from environmental remediation, the FAC can be used in other technological applications such as biomedical applications and thermal energy storage, etc.

## Figures and Tables

**Table 1:** Fourier transformed infrared spectroscopic vibrational bands of the different functional groups in FAC before and after the metal ion adsorption.

Assignment	FAC	FAC-Cr^6+^	FAC-Pb^2+^	FAC-Cd^2+^	FAC-Zn^2+^	Reference
**V (C-H)**	2815	2878	2793	2844	2830	[27,28,33]
**C=O**	1707	1720	1715	1725	1725	[27,28,33]
**N=O**	1581	1590	1587	1588	1590	[27,28,33]
	1520	1527	1525	1528	1525	[33,34]
**N-O**	1328	1331	1327	1327	1336	[33,34]
**C-N**	1250	1250	1252	1250	1256	[33,34]
**C-C**	1162	1182	1177	1173	1182	[27,33,34]
	1020	1026	1023	1020	1030	
	911	899	900	905	908	
	837	819	823	818	832	
**CH_2_ (rocking)**	735	733	730	735	734	

**Table 2:** Kinetic models and their detailed parameters.

Kinetic Model	Parameters
Pseudo-first-order	q_e_ = adsorption equilibrium q_t_ = adsorption at time t k_1_ = rate constant for pseudo-first-order ln = natural log
Pseudo-second-order	q_e_ = adsorption equilibrium q_t_ = Adsorption at time t k_2_ = Rate constant for Psudo second Order
Parabolic-Diffusion	M_o_ = q_e_ M_t_ = (q_e_ − q_t_)/q_e_ t^0.5^ = at half time K = the adsorption constant

**Table 3:** Correlation coefficients (R^2^) and constants for the Langmuir and Freundlich isotherms for Pb^2+^, Cd^2+^, Zn^2+^ and Cr^6+^ adsorption on FAC.

Metal Ion	Langmuir Isotherm			Freundlich Isotherm		
	q_e_ (mg/g)	b (L/mg)	R^2^	K_f_	n_f_	R^2^
Cd^2+^	38	0.04	0.82	0.95	0.45	0.96
Pb^2+^	40	0.03	0.84	0.32	1.04	0.97
Zn^2+^	37	0.04	0.93	0.29	1.60	0.96
Cr^6+^	40	0.04	0.85	0.64	0.71	0.96

## Data Availability

Not applicable.
